# Anti-tumor effect of Wasabi component, 6-(methylsulfinyl) hexyl isothiocyanate, against endometrial carcinoma cells

**DOI:** 10.1007/s12672-023-00617-2

**Published:** 2023-01-23

**Authors:** Motoki Ono, Tsutomu Miyamoto, Chiho Fuseya, Ryoichi Asaka, Hirofumi Ando, Yasuhiro Tanaka, Manaka Shinagawa, Yusuke Yokokawa, Hodaka Takeuchi, Akiko Horiuchi, Tanri Shiozawa

**Affiliations:** 1 Department of Obstetrics and Gynecology, Shinshu University School of Medicine, 3-1-1 Asahi, Matsumoto, 390-8621 Japan; 2 Horiuchi Ladies Clinic, 1-16-3 Tsukama, Matsumoto, 390-0821 Japan

**Keywords:** 6-(Methylsulfinyl) hexyl isothiocyanate, Wasabi, Endometrial carcinoma, Anti-tumor effect, Natural killer cell activity, Apoptosis

## Abstract

**Purpose:**

Wasabi is a traditional plant seasoning with an anti-septic function. Recent studies revealed several functions of Wasabi, such as anti-inflammation; however, the anti-tumor effect against endometrial carcinoma (EMC) cells has not been examined. In the present study, we investigated the anti-tumor effect of 6-(methylsulfinyl) hexyl isothiocyanate (6-MITC), a major chemical compound of Wasabi, against various EMC cell lines in vitro and in vivo.

**Methods:**

The effect of 6-MITC on cell viability was measured by the WST-1 assay in EMC and HUVEC cells. The impact of 6-MITC oral administration in nude mice was measured to assess the growth of the EMC xenograft and natural killer (NK) cell activity in the spleen.

**Results:**

The addition of 6-MITC suppressed the proliferation of EMC cells (Ishikawa, HEC265, HEC108, KLE, and HEC1B) dose-dependently, but not HUVEC cells. 6-MITC (5 µM) enhanced the cisplatin sensitivity of EMC cells. 6-MITC induced apoptosis in a dose-dependent fashion in EMC cells other than HEC1B cells and was associated with increased expression of cleaved-caspase3 and decreased expression of BCL2. Oral administration of 6-MITC (2 and 4 µmol/kg) to Ishikawa and HEC1B xenografting mice resulted in a reduced tumor volume compared with the control (P < 0.05, 4 µmol/kg). Immunohistochemical staining of resected tumors revealed increased expression of Ki-67 and reduced cleaved-caspase3. Furthermore, 6-MITC treatment enhanced NK cell activity, especially when administered before tumor xenografting.

**Conclusion:**

These results indicate that 6-MITC has a marked anti-tumor effect against EMC cells and a novel effect to enhance NK cell activity. These effects suggest the therapeutic potential of 6-MITC.

**Supplementary Information:**

The online version contains supplementary material available at 10.1007/s12672-023-00617-2.

## Introduction

The number of patients with endometrial carcinoma (EMC) is increasing persistently in Western countries and markedly in Japan [1]. The mainstay of treatment is surgery, with adjuvant chemotherapy such as carboplatin and paclitaxel [2]. The prognosis associated with early-stage EMC is generally favorable; however, that of advanced stages or recurrent cases is still poor [3]. Recently, several new drugs such as molecular targeted drugs and immune checkpoint inhibitors showed effectiveness for some patients [4, 5]; however, these agents have been reported to be associated with adverse effects [4, 5]. Therefore, the anti-tumor effects of natural compounds, which have been used for a long time, are expected as an alternative to these drugs or an additional option.

Six-(methylsulfinyl) hexyl isothiocyanate (6-MITC) is a natural compound extracted from the rhizome of Wasabi (also known as Japanese horseradish or *Wasabia japonica*) [6] and shows a unique spicy flavor. Due to this flavor, Wasabi has been used as an ingredient and seasoning in Japan for over 1000 years. Especially, Wasabi has long been known to have an anti-septic function [7]. Therefore, Wasabi has been eaten with raw fish in Japan for dietary hygiene. Because our research facility is located in Nagano Prefecture, Japan, which is well-known as an area that produces Wasabi, we focused on 6-MITC, a major chemical compound of Wasabi [8]. 6-MITC has been reported to exhibit various bioactivities such as anti-inflammatory [9], antioxidative [10], and glucose tolerance improvement [11, 12], and has been suggested to show anti-tumor effects against melanoma [13], pancreas [14], breast [15], colorectal [16, 17], and hematological tumors [18]. However, there is no report on its cytotoxic effects against EMC.

In this study, we demonstrated that 6-MITC has anti-tumor effects on several EMC cell lines and investigated the anti-tumor mechanisms. In addition, we showed that 6-MITC-induced growth suppression of tumors was associated with the activation of innate immunity in vivo. These results indicate the therapeutic potential of 6-MITC against EMC.

## Materials and methods

### Isothiocyanates

We used three types of isothiocyanates: 6-MITC (ab142898), 4-(methylsulfinyl) butyl isothiocyanate (sulforaphane, 4-MITC) (4478-93-7), and allyl isothiocyanate (AITC) (016-01463), which were purchased from Abcam (Cambridge, UK), Cayman Chemical (Ann Arbor, MI, USA), and FUJIFILM Wako Pure Chemical (Osaka, Japan), respectively. We immediately used them after dilution in dimethyl sulfoxide for the cell viability assay or used 6-MITC for the animal study after dilution in distilled water. We used AITC and 4-MITC as positive controls for 6-MITC in cell viability experiments because these isothiocyanates (ITCs) are known to show anti-tumor activity [19–21].

### Cell cultures and reagents

The EMC cell line, Ishikawa 3-H-12 (Ishikawa) was a kind gift from Dr. Nishida (Kasumigaura Medical Center, Ibaraki, Japan) who established this cell line. HEC108 and HEC265 cells were supplied by JCRB Cell Bank (Osaka, Japan). HEC1B and KLE cells were purchased from ATCC (Manassas, VA, USA). The natural killer (NK) cell-sensitive mouse lymphoma cell line Yac1 was subdivided from RIKEN BioResource Center, and kindly donated to us by Prof. Taki (Department of Molecular and Cellular Immunology, Shinshu University School of Medicine). As normal non-tumor cells, Human Umbilical Vein Endothelial Cells (HUVECs) and the immortalized human uterine endometrial progenitor cell line (EM-E6/E7-hTERT2) were from ATCC and JCRB Cell Bank, respectively. According to the recommended protocol, cells were maintained in a medium with fetal bovine serum and incubated at 37 °C in air containing 5% CO_2_. The number of passages of cell lines used in this study was less than 20.

### WST-1 assay

According to the manufacturer's instructions, we assessed cell viability using the WST-1 reagent (Roche Diagnostics, Basel, Switzerland). Briefly, 2000 cells/well were plated onto 96-well plates and incubated for 24 h. Then, the cells were treated with 6-MITC for 48 h, and the medium was changed to a phenol-red-free one containing 10% WST-1 reagent. After 2.5 h of incubation, we measured the absorbance at a 450-nm wavelength using Synergy HT Microplate Reader (Bio-Tek Instruments, Winooski, VT, USA). We obtained each result from two or three independent experiments performed in octuplicate for every condition.

For IC50 calculation, we treated endometrial cancer cell lines with 6-MITC concentrations of 0, 2, 4, 8, 10, 15, 20, 25, 30, and 40 μM for 48 h and measured cell viability by the WST-1 assay. Then, using ImageJ (public domain: https://imagej.nih.gov/ij/index.html), we calculated IC50 values of 6-MITC for each cell line by a 4-parameter logistic curve (Rodbard): y = ((a − d)/(1 + (x/c)^b^)) + d.

### Western blotting

Cells were lysed in an RIPA lysis buffer (Millipore Corporation, Billerica, MA, USA) containing the protease inhibitor cocktail Complete Mini (Roche Diagnostics). Cell lysates were centrifuged at 15,000×*g* for 5 min, and then we collected the supernatant and measured the protein concentration. Ten micrograms of protein of each sample were electrophoresed on NuPAGE Bis–Tris Gels in NuPAGE MES SDS Running Buffer and transferred to PVDF blotting membranes (Thermo Fisher Scientific, Wilmington, DE, USA). Membranes were blotted with the following primary antibodies: rabbit polyclonal anti-bcl2 antibody (sc-492, Santa Cruz Biotechnology, Dallas, TX, USA), rabbit polyclonal anti-cleaved caspase3 antibody (#9661, Cell Signaling Technology, Danvers, MA, USA), and rabbit polyclonal anti-GAPDH antibody (GTX100118, GeneTex, Irvine, CA, USA) at 4 °C overnight and then with an anti-rabbit IgG, horseradish peroxidase-linked species-specific whole antibody (Amersham, Piscataway, NJ, USA) at room temperature for 1 h. Bound antibodies were then visualized using the ECL Western blot detection reagent (Amersham).

### Fluorescent immunostaining of apoptotic cells

We used an Apoptotic/Necrotic/Healthy cell detection kit (PK-CA707-30018, PromoCell, Heidelberg, Germany) to detect apoptotic cells according to the manufacturer's instructions. This method can simultaneously measure the four types of cells by triple synchronous fluorescence staining. Green indicates early-stage apoptotic cells stained by Annexin V. Red denotes necrotic cells stained by Ethidium Homodimer III. Yellow shows late-stage apoptotic cells stained by both red and green fluorescence. Blue represents living cells stained by Hoechst 33342. In three independent experiments, we measured the percentage of apoptotic cells in 100 (40 µM of 6-MITC) to 1000 cells.

### Animal studies

Six-week-old female BALB/C nu/nu mice were purchased from The Jackson Laboratory Japan (Yokohama, Japan).

For the dose-intensity experiment, mice were subcutaneously transplanted with 1.0 × 10^7^ Ishikawa and 5.0 × 10^6^ HEC1B cells in the right and left lower backs, respectively. After implantation, mice were divided into three groups (6 mice for each): the control group received 200 μL/day of distilled water, and the 2 µmol/kg- and 4 µmol/kg-administered groups received 2 and 4 µmol/kg/day of 6-MITC diluted in 200 µL of distilled water, respectively, by daily oral administration for 5 weeks.

For the mouse experiment to investigate the effect of 6-MITC on NK cell activity, mice were divided into three groups (5 mice for each). The "Preceding" group was orally administered 4 µmol/kg/day of 6-MITC daily for 4 weeks before and 5 weeks after implantation. The "Treatment" group was administered 200 μL/day of distilled water before implantation and 4 µmol/kg/day of 6-MITC after implantation. The "Control" group received 200 μL/day of water throughout the experimental period. All mice were subcutaneously implanted with 1.0 × 10^7^ Ishikawa and 3.0 × 10^6^ HEC1B cells in the right and left lower back, respectively.

We calculated the xenograft tumor volume weekly in both experiments using the following formula: tumor volume = 1/2 × (long diameter) × (short diameter)^2^. We euthanized mice at the endpoint of experiments, removed all xenograft tumors, and measured the tumor volume and weight. In the latter experiment, spleens were removed and used for the subsequent NK-cell activity assay. We recorded each group's average water intake and body weight to evaluate the safety of 6-MITC oral administration.

The Committee for Animal Experiments of Shinshu University reviewed and approved all mouse xenograft experiments (Approval Number: 300105), in which during the observation period, mice with tumors exceeding 17 mm in diameter, with weight loss of more than 20% or with markedly decreased activity should be euthanized from a humane point of view.

### Immunohistochemistry

Four-micrometer-thick sections of formalin-fixed and paraffin-embedded tissues were deparaffinized, rehydrated, and boiled in a microwave oven for 30 min with 0.01 M citrate buffer (pH 6.0). They were treated with 0.3% hydrogen peroxide for 15 min to inhibit endogenous peroxidase activity and incubated with primary antibodies at 4 °C overnight. Rabbit polyclonal anti-cleaved caspase-3 antibody (#9661, Cell Signaling Technology) and rabbit monoclonal anti-Ki-67 antibody (GTX16667, GeneTex) were primary antibodies. After washing with phosphate-buffered saline, the sections were incubated with horseradish peroxidase-conjugated secondary antibodies using Histofine MAX-PO (Nichirei, Tokyo, Japan) for 60 min at room temperature and stained with diaminobenzidine in 0.15% hydrogen peroxide. Counterstaining with hematoxylin was then performed. We calculated the value of % -positive cells from the number of positive cells in 1000 tumor cells in each xenograft.

### NK-cell activity assay

Each removed spleen was passed through a 40-μm nylon cell strainer and resuspended in Hanks balanced saline solution (HBSS). After the erythrocyte-lysing process, we collected the spleen-derived cells with NK cells. Then, we measured the cytotoxicity of the NK cells in the spleen-derived cells using LDH Cytotoxicity Detection Kit (MK401, TaKaRa Bio, Kusatsu, Japan) according to the manufacturer's instructions. In this method, NK cell activity is evaluated by measuring lactate dehydrogenase (LDH) activity released from target cells, the NK-cell-sensitive mouse lymphoma cell line Yac1, when the NK cells in spleen-derived cells damage Yac1 cells.

### Statistical analysis

We conducted statistical analyses with the Mann–Whitney U test for two-group comparisons or Kruskal–Wallis test followed by Scheffe's test for multi-group comparisons using SPSS version 24 (IBM, Armonk, NY, USA). A p-value less than 0.05 was significant.

We confirmed that the post-hoc powers of all experiments were 0.80 or more and sufficient N numbers using SPSS.

## Results

### Effect of 6-MITC on viability of EMC cells

**Fig. 1:**
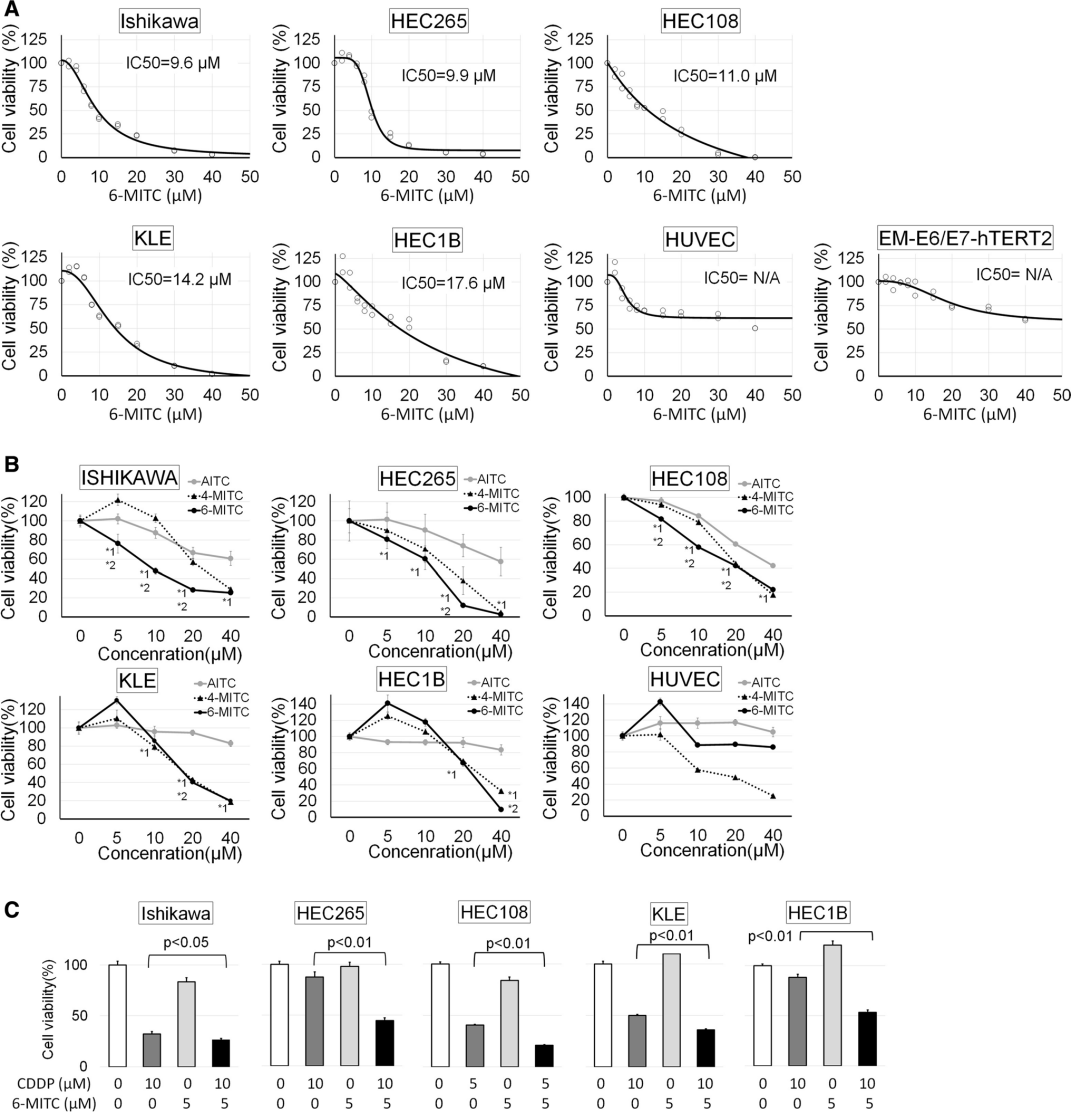
**A** The 6-MITC dose-dependent cell viability curve. The cell viability at each dose was measured using the WST-1 assay in EMC cell lines, Ishikawa, HEC265, HEC108, KLE, and HEC1B, and normal cell lines, HUVECs, and EM-E6/E7-hTERT2. Cell viability curves were drawn using the 4-parameter logistic method. The calculated IC50 values of each cell line are expressed in the Figure. On the other hand, the effects of 6-MITC for HUVECs and EM-E6/E7-hTERT2 were too weak to calculate IC50 values. **B** The effects of 6-MITC, AITC, and 4-MITC on cell viability were examined using the WST-1 assay in EMC cell lines and HUVECs. 6-MITC significantly reduced cell viability compared with AITC and 4-MITC, dose-dependently, especially in Ishikawa, HEC265, and HEC108 cells. In contrast, 5 µM of 6-MITC significantly increased cell viability in KLE and HEC1B (p < 0.01). Unlike 4-MITC, 6-MITC and AITC did not reduce cell viability to below 80%. *1: significant reduction (p < 0.05) of cell viability with each dose of 6-MITC compared with 0 µM of 6-MITC and the same dose of AITC. *2: significant reduction (p < 0.05) of cell viability with each dose of 6-MITC compared with 0 µM of 6-MITC and the same dose of 4-MITC. **C:** Effect of 5 µM 6-MITC addition on CDDP sensitivity in EMC cell lines using the WST-1 assay. The addition of 5 µM 6-MITC alone did not sufficiently reduce cell viability but significantly enhanced CDDP sensitivity. The effect was marked on HEC265 and HEC1B cells, which are less sensitive to CDDP. Cell viability is the percentage relative to that at 0 µM. Graphs indicate mean values, and error bars indicate standard deviations. These results were obtained from two (**A**) or three (**B** and **C**) independent experiments performed in octuplicate for every condition

First, we examined the effect of 6-MITC on cell viability using EMC cell lines and compared the effect with the solvent only (0 µM 6-MITC) as a negative control and with structurally similar compounds, AITC and 4-MITC, as positive controls. The WST-1 assay revealed that 6-MITC inhibited the proliferation of Ishikawa, HEC265, HEC108, KLE, and HEC1B cells dose-dependently, with IC50 values of 9.6, 9.9, 11.0, 14.2, and 17.6 μM, respectively (Fig. 1A). On the other hand, the effect on cell viability of normal non-tumor cells, HUVECs and EM-E6/E7-hTERT2, was so small that we could not calculate IC50 values (Fig. 1A). In addition, the growth-inhibitory effect of 6-MITC was stronger than that of AITC (Fig. 1B). A total of 5 µM of 6-MITC significantly suppressed the viability of Ishikawa, HEC265, and HEC108 cells but significantly increased that of KLE and HEC1B cells, indicating that the effect of 6-MITC on proliferation may differ among cell lines (Fig. 1B). The effect of 6-MITC was similar to 4-MITC, but 6-MITC significantly reduced cell viability compared with 4-MITC at low doses (5 and 10 µM) in Ishikawa and HEC108 cells (Fig. 1B). On the other hand, both AITC and 6-MITC only suppressed the proliferation of less than 20% of HUVEC cells (Fig. 1B).

### Effect of 6-MITC on cisplatin (CDDP) chemosensitivity

In order to investigate the effect of 6-MITC on CDDP sensitivity, we cultured EMC cells treated with solvent only as a negative control, with 5 or 10 µM CDDP, with 5 µM 6-MITC, and with both CDDP and 6-MITC, and then measured cell viability by the WST-1 assay. Although 5 µM 6-MITC showed little cytotoxicity, it significantly enhanced the CDDP sensitivity of all EMC cell lines, especially HEC265 and HEC1B cells, which were less sensitive to CDDP (Fig. 1C).

### Effect of 6-MITC on apoptosis induction in EMC cells

**Fig. 2:**
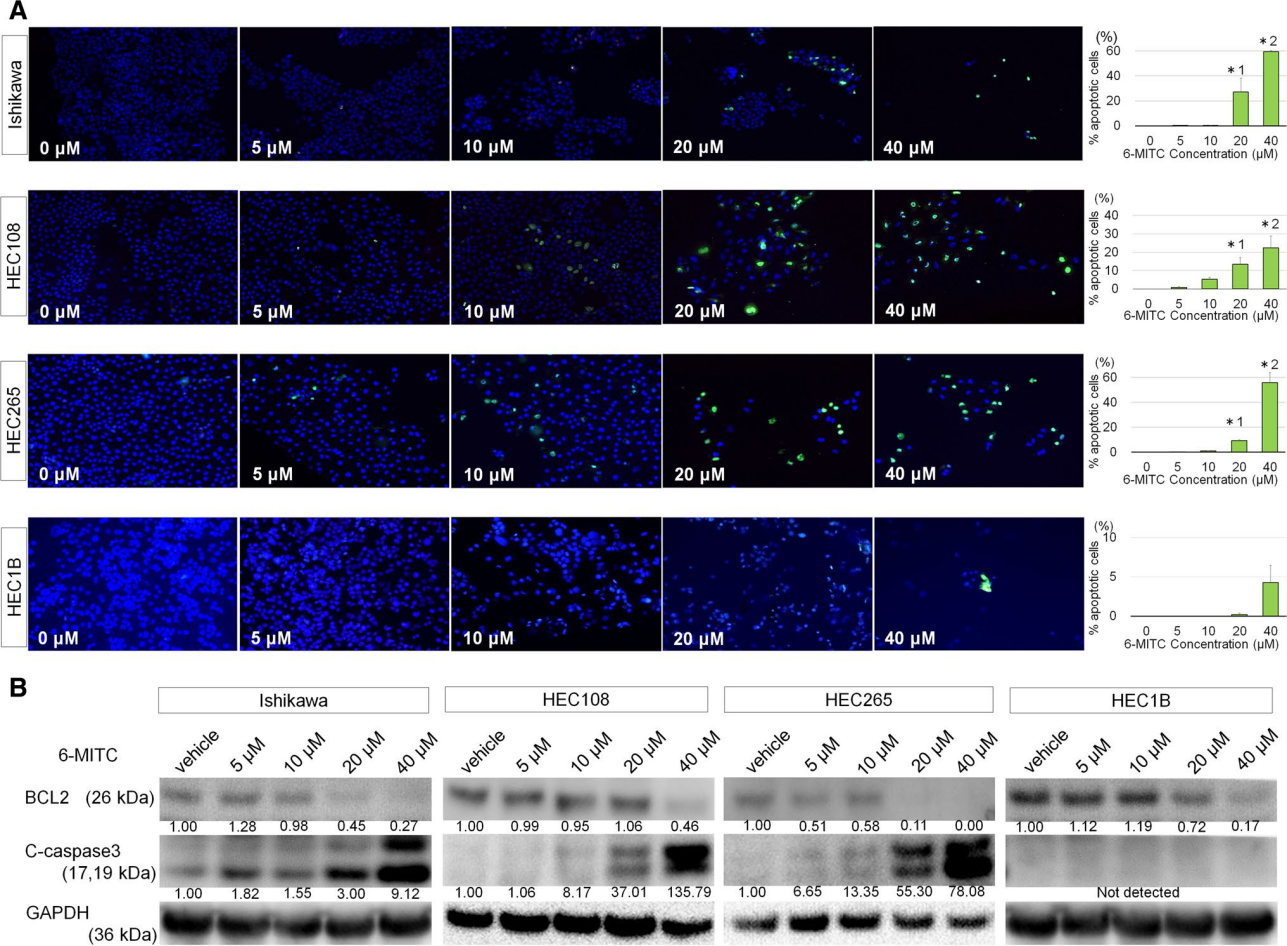
**A** Effect of 6-MITC on apoptosis in EMC cell lines. Pictures show apoptotic cells with green or yellow fluorescence. Graphs indicate mean values of the percentage of apoptotic cells of 100 (40 µM of 6-MITC) to 1000 cells in three independent experiments, and error bars indicate the standard deviations. 6-MITC induces apoptosis in Ishikawa, HEC108, and HEC265 cells, dose-dependently. However, 6-MITC treatment does not increase apoptosis in HEC1B cells. *1 and *2 indicate significant differences compared with 0 µM, at p < 0.05 and p < 0.01, respectively. **B** Western blotting reveals that 6-MITC reduces the expression of anti-apoptotic proteins, BCL2, in EMC cell lines. Every numerical value below the photographs is expressed as a ratio to the vehicle after quantifying the band density by densitometry and correcting it with GAPDH. 6-MITC enhances the expression of the proapoptotic protein c-caspase3 in Ishikawa, HEC108, and HEC265 cells but not in HEC1B cells

To examine the involvement of apoptosis in 6-MITC-induced suppression of cell proliferation, we performed Annexin V staining of EMC cells treated with various concentrations of 6-MITC and measured the percentage of Annexin V-positive apoptotic cells. Apoptotic cells were significantly increased in Ishikawa, HEC108, and HEC265 cells treated with 20 µM or more of 6-MITC (Fig. 2A). In contrast, enhanced apoptosis was not shown in HEC1B cells with any concentration of 6-MITC (Fig. 2A). Western blotting revealed that cleaved-caspase3 (c-caspase3), a marker of apoptosis, was up-regulated in Ishikawa, HEC108, and HEC265 cells by 6-MITC treatment dose-dependently, but not in HEC1B cells. BCL2, an anti-apoptotic protein, was down-regulated dose-dependently (Fig. 2B).

### Effect of 6-MITC on the tumor growth of EMC xenografts

**Fig. 3:**
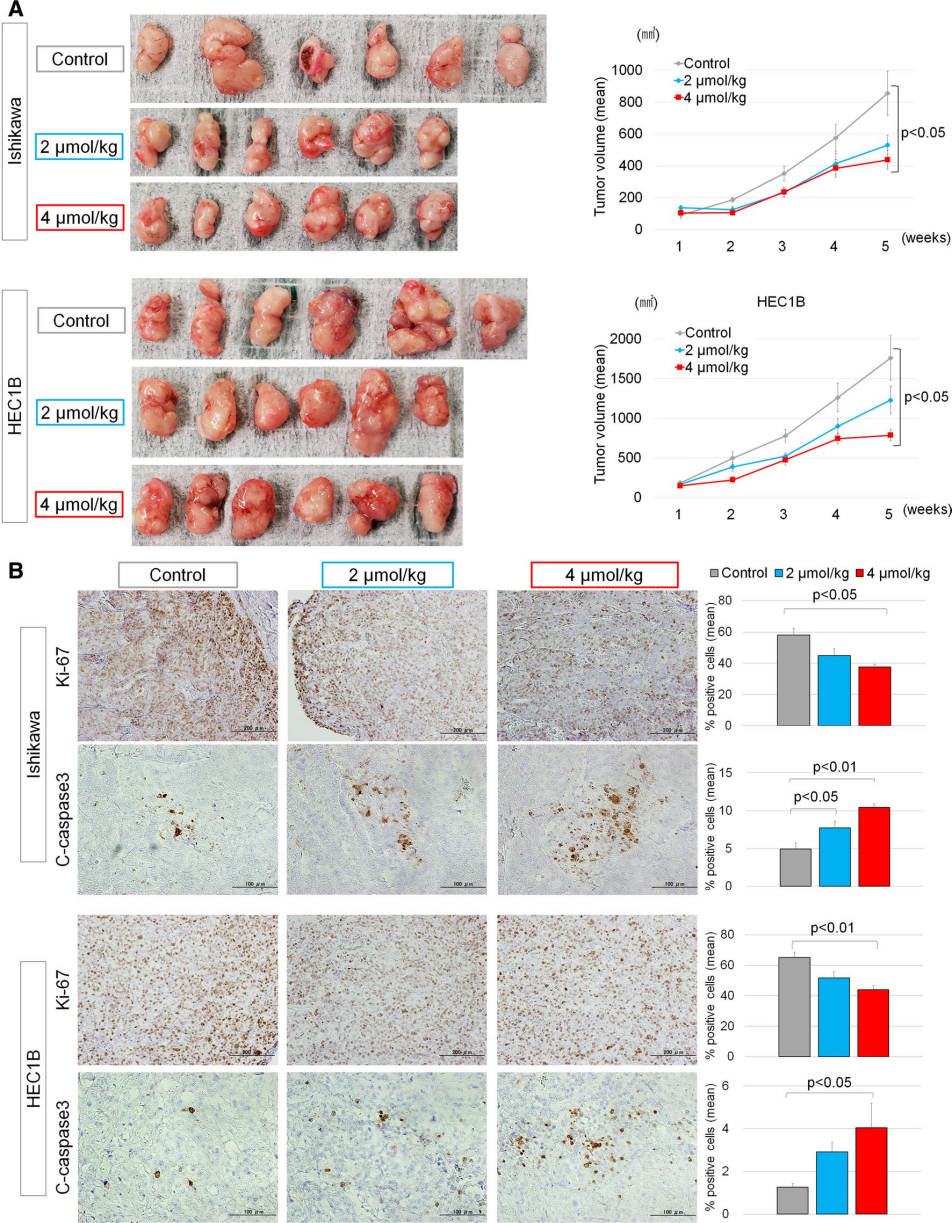
**A** Effect of daily 6-MITC oral administration on Ishikawa and HEC1B xenograft tumor growth in nude mice. Xenografted mice were divided into three groups (Control, 2 µmol/kg-, and 4 µmol/kg-administered groups); each group had six mice. Pictures are at the same scale and show each tumor removed from each mouse after euthanization at 5 weeks. Graphs indicate mean values, and error bars indicate standard deviations. At 5 weeks, the tumor volume of the 4 µmol/kg-administered group was significantly decreased compared with the control group for both Ishikawa (P = 0.01) and HEC1B (P = 0.02) cells. **B** Immunohistochemical (IHC) results of the removed xenografts for Ki-67 and c-caspase3 are shown in pictures, and the average values of %-positive cells are indicated in graphs with standard deviations (error bars). 6-MITC administration reduced the expression of Ki-67 and enhanced c-caspase3 in both Ishikawa and HEC1B cells

We investigated the antitumor effect of 6-MITC administration against subcutaneous xenografts of Ishikawa and HEC1B cells in BALB/C nude mice. At 5 weeks, some xenografts exceeded 17 mm in diameter. Hence, we euthanized mice at that time according to the experimental plans approved by the Committee for Animal Experiments of Shinshu University. After 5 weeks of 6-MITC oral administration, the tumor volume of the 4 µmol/kg-administered group was significantly decreased compared with the control group, in both Ishikawa (P = 0.01) and HEC1B (P = 0.02) cells (Fig. 3A, Supplementary Fig. A). The tumor volume of the 2 µmol/kg-administered group was also smaller than in the control, but this was not significant. Bodyweight gain and water intake volume were not different among the three groups (data not shown), suggesting no severe adverse effect of the tested 6-MITC doses.

Immunohistochemical staining (IHC) of the removed tumors from each group revealed that the ratio of Ki-67-positive cells was significantly decreased, and the percentage of c-caspase3-positive cells was significantly increased in the 4 µmol/kg-administered group in both cell lines (Fig. 3B). These IHC results showed that 6-MITC treatment induced apoptosis of HEC1B cells as well as Ishikawa cells, differing from the results of in vitro experiments.

### Effect of 6-MITC on activation of NK cells in HEC1B xenograft-bearing mice

**Fig. 4:**
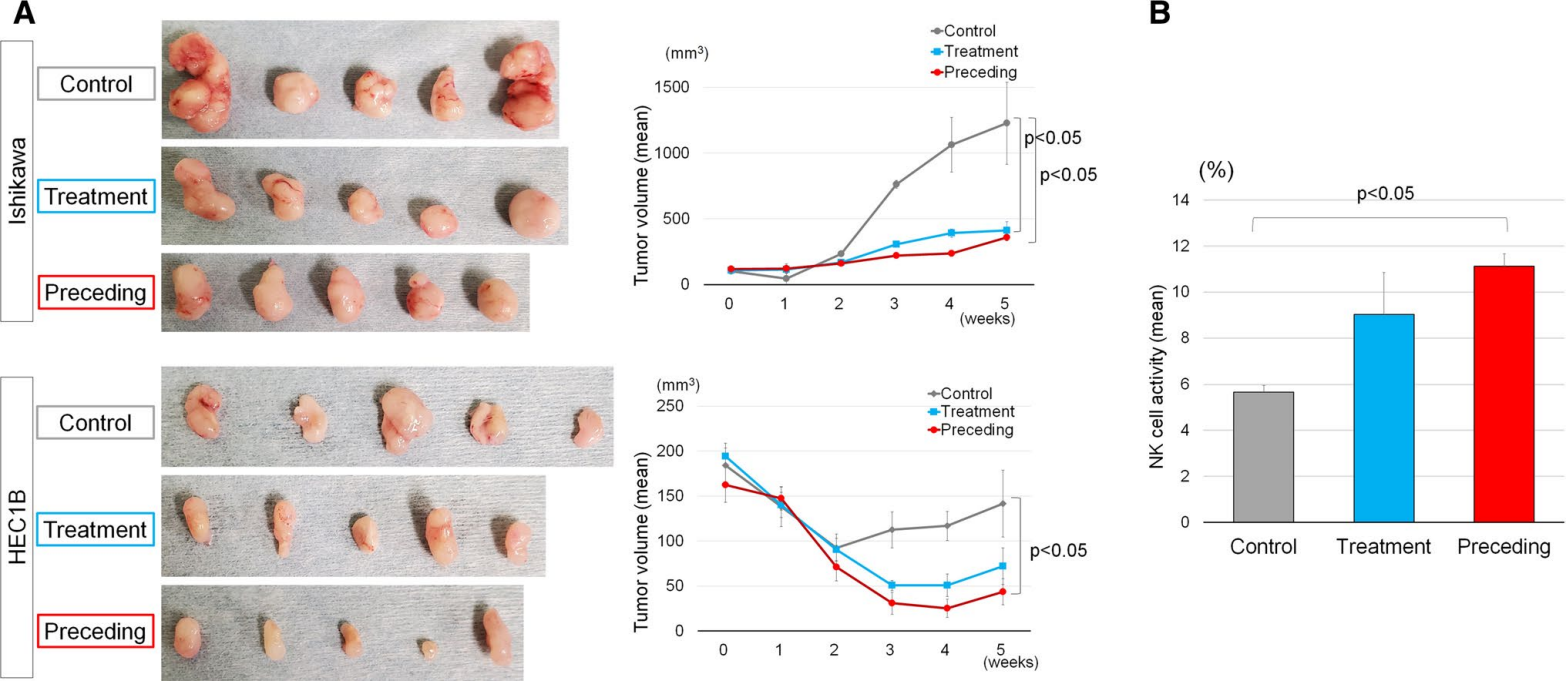
Effect of daily 6-MITC oral administration on Ishikawa and HEC1B xenograft tumor growth and NK cell activity in nude mice. Xenografted mice were divided into three groups (Control, Treatment, and Preceding groups); each group had five mice. Oral administration of 6-MITC was started from xenografting in the "Treatment" group and 4 weeks before xenografting in the "Preceding" group. **A** Pictures are at the same scale and show each tumor removed from each mouse after euthanization at 5 weeks. 6-MITC oral administration significantly reduced the tumor volume in both "Treatment" and "Preceding" groups compared with the Control group. **B** 6-MITC oral-administration enhanced NK-cell activity in xenografted nude mice, with significance in the "Preceding" group. Graphs indicate mean values, and error bars indicate standard deviations

Since nude mice lack T cells but possess NK cells, we hypothesized that the 6-MITC-induced apoptosis of HEC1B cells in the nude mouse model was mediated via NK-cell activation. To test this hypothesis, we prepared two mouse groups: "Preceding group": daily oral administration of 4 µmol/kg 6-MITC started 4 weeks before xenografting, and "Treatment group": the same oral dose of 6-MITC started after xenografting. We euthanized mice at 5 weeks because some xenografts exceeded 17 mm in diameter. We compared the growth of xenografts and NK-cell activation of each mouse group with the control group. The results indicated that the volumes of xenografts were significantly smaller in both preceding and treatment groups than in Control. However, there was no difference between the Preceding and Treatment groups (Fig. 4A, Supplementary Fig. B). An NK-cell cytotoxicity assay revealed that NK-cell activity was significantly higher in the Preceding group than in the Control group (Fig. 4B). NK-cell activity in the Treatment group was also higher than in the Control group, but this was not significant. This suggested that 6-MITC-induced apoptosis was evoked by activating NK cells.

## Discussion

An important group of compounds that have chemopreventive properties is organosulfur compounds such as isothiocyanates (ITCs), as represented by allyl isothiocyanate (AITC) in black mustard, benzyl isothiocyanate (BITC) in papaya, phenylhexyl isothiocyanate (PITC) in cabbage, 4-(methylsulfinyl) butyl isothiocyanate (sulforaphane, 4-MITC) in broccoli, and 6-(methylsulfinyl) hexyl isothiocyanate (6-MITC) in Wasabi [20, 21]. The present study demonstrated that 6-MITC suppressed the proliferation of EMC cells in vitro and in vivo, associated with increased apoptosis via up-regulation of c-caspase3. This is the first report of the anti-tumor effect of 6-MITC against EMC.

The anti-tumor effects of 6-MITC have been reported against other tumors such as breast and skin cancer [13, 15], pancreas cancer [14], colorectal cancer [16, 17], and hematological tumors [18, 22]; however, the effects vary among cell types. Nomura T et al. found that the growth-inhibitory effect of 6-MITC was significantly stronger than that of other ITCs including sulforaphane, and reported that breast carcinoma (MCF-7) and melanoma (LOX-IMV) cell lines showed high sensitivity to 6-MITC, *ie*., 6-MITC completely suppressed MCF-7 and LOX-IMV at 10^–7^ and 10^−6^ M, respectively [13]. In addition, IC50 of one melanoma cell line (LOX-IMV) for 6-MITC was as low as 0.3 µM [13]. The half maximal inhibitory concentrations of 6-MITC at 24 and 48 h for K562 human chronic myeloid leukemia cells was estimated to be approximately as 13.0 and 7.8 µM, respectively [18]. The IC50 value calculated by interpolation was 8.65 μM for leukemia Jurkat cells, and 16 μM for HL-60 cells (24 h) [22]. The IC50 value for the pancreatic cell line PANC-1 was approximately 10 ~ 20 µM [14]. In contrast, the effect of original 6-MITC against oral cancer was very weak [23]. In the present study, IC50 values of five EMC cell lines were 9.6, 9.9, 11.0, 14.2, and 17.6 μM, respectively. Therefore, the sensitivity of EMC cells to 6-MITC is largely at an “intermediate” level. It is noteworthy that the effect of 6-MITC on the proliferation of HUVECs and EM-E6/E7-hTERT2 cells was too low to calculate IC50 values. In addition, oral administration of 6-MITC (4 µmol/kg/ day) suppressed the growth of mouse xenografts without apparent adverse effects. The dosage of 6-MITC used in mouse experiments is considered tolerable for an adult human [24]. In addition, this study indicated that 6-MITC enhanced the sensitivity to CDDP, suggesting the merit of the supplementary use of 6-MITC with conventional chemotherapy.

The present study shows the induction of apoptosis by 6-MITC in EMC cells. On the other hand, cell viability was significantly reduced in HEC1B without increased expression of Annexin V or cleaved-caspase3, suggesting an anti-tumor effect other than apoptosis induction. Although apoptosis, necrosis, and G2/M arrest induced by 6-MITC have been reported in many articles, reports suggesting the mechanisms have been limited. One study reported the involvement of the ELK1/CHOP/DR5 pathway via ERK1/2 activation in G2/M arrest of colorectal cancer cells [17]. Another group demonstrated that 6-MITC induced autophagy via AMPK activation, but it was not associated with G2/M arrest [18]. Also, 6-MITC decreased nuclear expressions of NF-κB and phospho-Akt, suggesting an association with apoptosis in mouse xenografts [15]. The 6-MITC-induced apoptosis has been reported not to be associated with p53 activation [16]. In addition, a similar compound, 4-MITC, known as sulforaphane, has been reported to activate some signaling pathways, such as Nrf2, by epigenetic action targeting DNA methyltransferases, histone deacetylases (HDACs), and noncoding RNAs, resulting in anti-tumor activity [25].

We observed that a low dose of 5 µM 6-MITC unexpectedly enhanced the cell viability of KLE and HEC1B (Fig. 1B). Although the reason is unknown, a similar phenomenon in which cytotoxic drugs and radiation at low doses actually stimulate cell survival is often observed and termed 'hormesis' [26]. Since it has been reported that 6-MITC has beneficial effects on cell survival, such as antioxidative effects [10], it is conceivable that 6-MITC enhances survival at low doses of some cell lines.

Interestingly, this study demonstrated that 6-MITC enhanced NK-cell activity, an innate immune system in nude mice. Similar 6-MITC actions have never been reported. Although the mechanism has not fully been elucidated, sulforaphane (4-MITC) has also been reported to activate the NK-cell function [27, 28]. Since NK cells have been reported to induce cell death by apoptosis and necrosis [29], activated NK cells may assist in the induction of apoptosis by an extrinsic mechanism. Since athymic nude mice lack T cells, including helper T-cell associated maturation of B cells, in this study, we focused on NK cells in the innate immune system, which remain in nude mice and potentially induce apoptosis in HEC1B xenograft cells. Our next task is to clarify whether 6-MITC affects lymphocyte activity other than NK cells.

EMC has unique characteristics. First, EMC, is a steroid hormone-dependent tumor like breast cancer. Especially, chronic low estrogen, the so-called un-opposed estrogen milieu, is closely involved in its carcinogenesis. Second, a large-scale meta-analysis and population-based study described a strong correlation between the body-mass index and EMC incidence [30, 31]. Metabolic syndrome conditions such as diabetes have been reported as risk factors for EMC [32]. Several dietary habit factors associated with EMC incidence have also been reported. For example, a high glycemic load and Western-style dietary intake may increase the incidence of EMC. In contrast, coffee and fiber intake potentially reduce the risk of EMC [32]. In Japan, although the increase in obesity rates among women has been low for several decades [33], the EMC incidence has increased significantly (Source: Center for Cancer Control and Information Services, National Cancer Center, Japan. https://ganjoho.jp/reg_stat/index.html last visited on Jan. 14, 2022), which may be due to the change from former Japanese dietary habits to a Western style. Therefore, dietary habits are closely involved with endometrial carcinogenesis. This is supported by studies showing that weight reduction is effective to prevent the occurrence of EMC [34, 35]. In addition, EMC has been reported to contain frequent microsatellite instability (MSI) [36, 37]. MSI leads to many gene mutations, which eventually generate abundant neo-antigens on the cell surface, and these antigens are potential targets for local immune cells. Thus, the MSI-induced high immunogenicity of EMC suggests marked vulnerability of the local immune system such as NK cells [36–38]. Collectively, genesis and development of EMC can be influenced by extrinsic, environmental factors. Therefore, unique Japanese foods such as Wasabi may prevent EMC. Actually, Yano T et al. reported that 6-MITC prevented the genesis of lung cancer in mice [39].

In conclusion, the present study demonstrated the proapoptotic and anti-tumor effects of 6-MITC on EMC cells. 6-MITC administration suppressed tumor growth in mouse xenograft EMC tumors, potentially due to NK-cell activation. Further studies are warranted to clarify the effects of 6-MITC on the immune system.

## Supplementary Information

Additional file 1 (JPG 1669 KB)

Additional file 2 (JPG 568 KB)

Additional file 3 (JPG 889 KB)

Additional file 4 (JPG 953 KB)

## Data Availability

All data generated or analyzed during this study are included in this published article and its supplementary information files.
