# Supplementary figures and images for: Anti-tumor effect of Wasabi component, 6-(methylsulfinyl) hexyl isothiocyanate, against endometrial carcinoma cells
Source: Discov Oncol. 2023 Jan 23;14:9. doi: 10.1007/s12672-023-00617-2 (PMC9871149; doi:10.1007/s12672-023-00617-2)

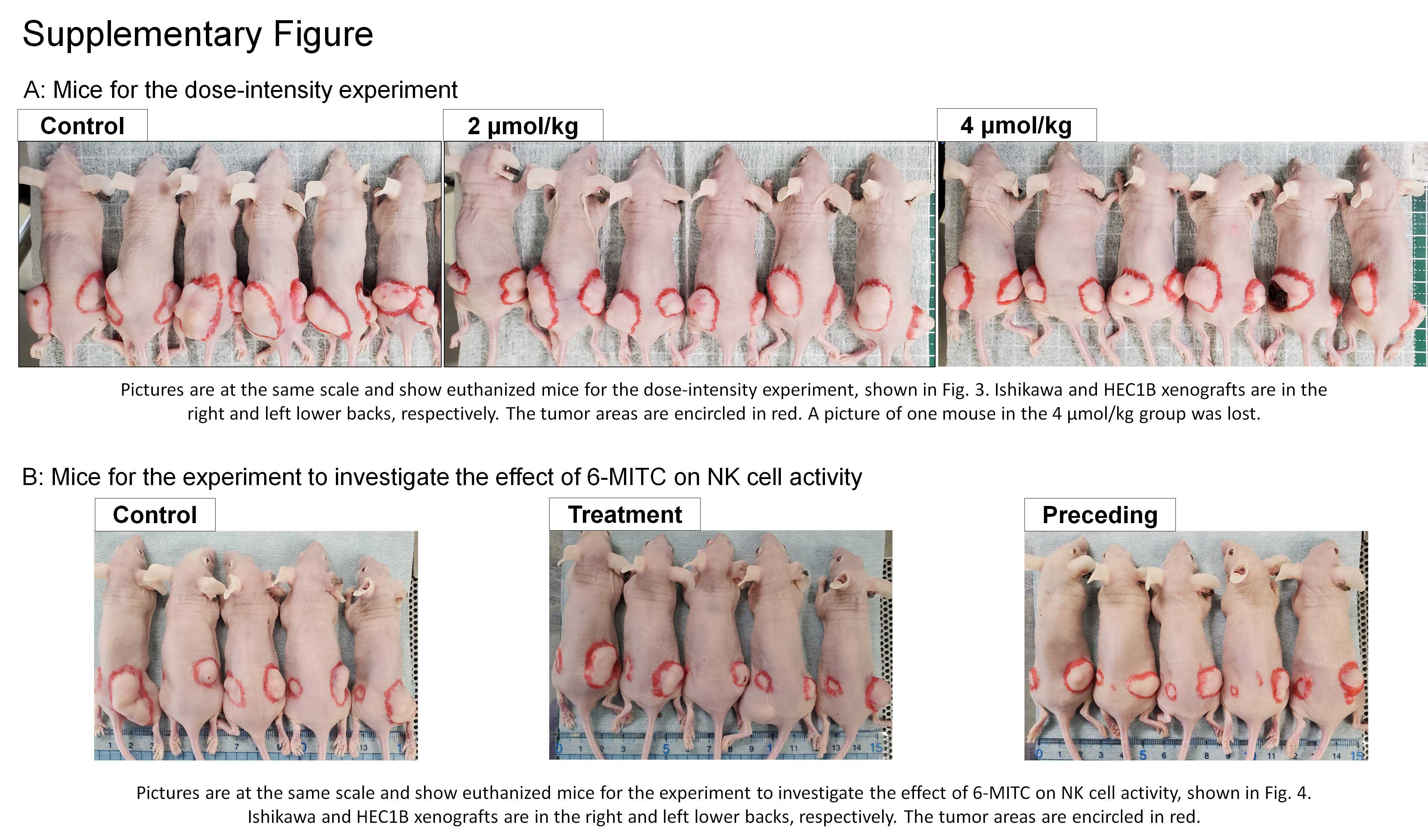

Supplement: Supplementary file 1 — Additional file 1 (JPG 1669 KB) [file 12672_2023_617_MOESM1_ESM.jpg]

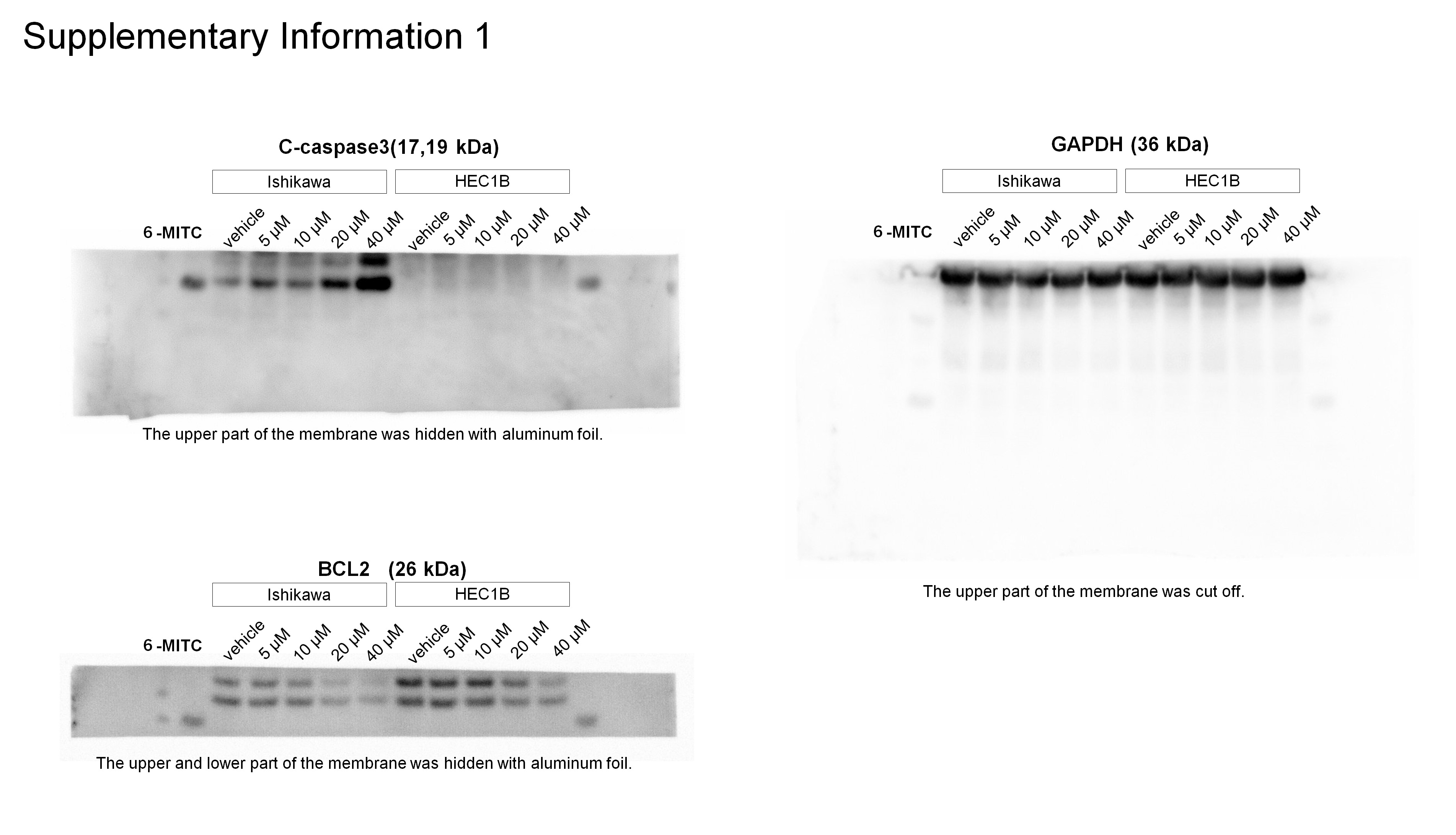

Supplement: Supplementary file 2 — Additional file 2 (JPG 568 KB) [file 12672_2023_617_MOESM2_ESM.jpg]

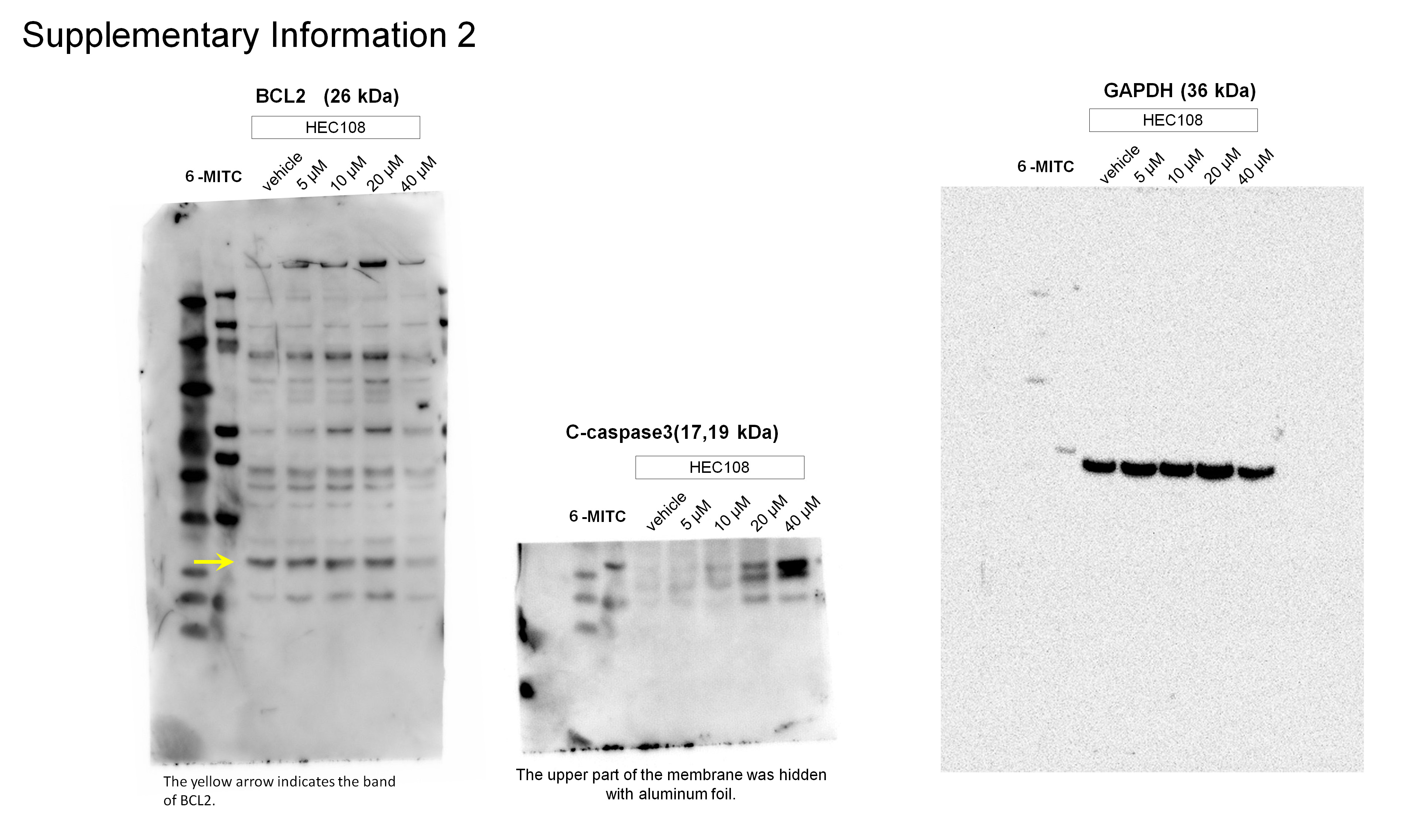

Supplement: Supplementary file 3 — Additional file 3 (JPG 889 KB) [file 12672_2023_617_MOESM3_ESM.jpg]

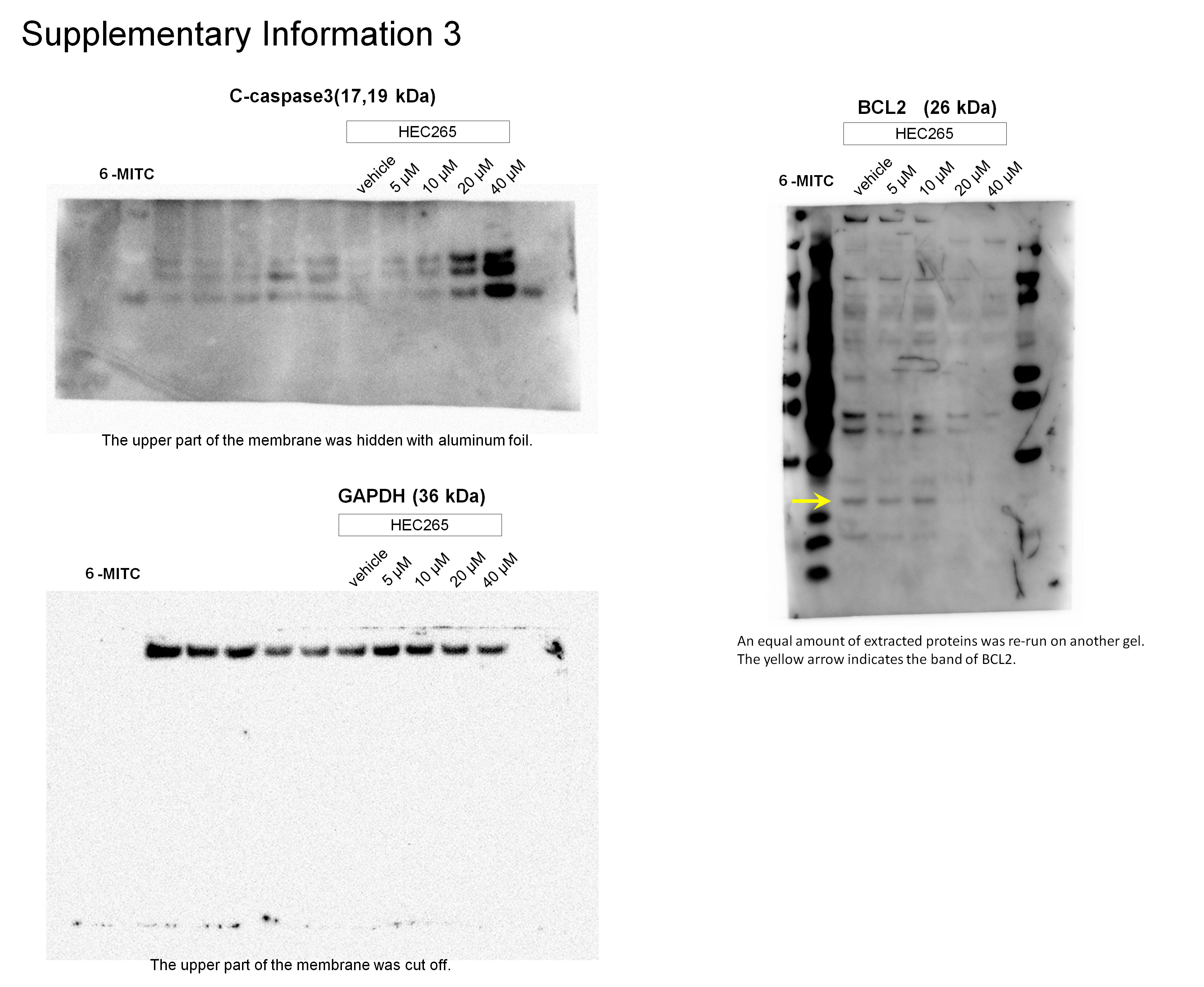

Supplement: Supplementary file 4 — Additional file 4 (JPG 953 KB) [file 12672_2023_617_MOESM4_ESM.jpg]
